# Predominant Tuberculosis Spoligotypes, Delhi, India

**DOI:** 10.3201/eid1006.030575

**Published:** 2004-06

**Authors:** Urvashi Balbir Singh, Naga Suresh, N.Vijaya Bhanu, Jyoti Arora, Hema Pant, Sanjeev Sinha, Ram Charan Aggarwal, Sushma Singh, Jitendra Nath Pande, Christophe Sola, Nalin Rastogi, Pradeep Seth

**Affiliations:** * All India Institute of Medical Sciences, New Delhi, India;; † Institut Pasteur de Guadeloupe, Pointe-à-Pitre, Guadeloupe

**Keywords:** *Mycobacterium tuberculosis*, spoligotyping, spoligotype, cluster, direct repeat locus, dendrogram, prevalence, spoligo database, transmission, CAS-I family, Beijing genotype

## Abstract

One hundred five *Mycobacterium tuberculosis* clinical isolates from the Delhi area were typed by spoligotyping; 45 patterns were identified. Comparison with an international spoligotype database showed type 26, Delhi type (22%), type 54 (12%), and type 1, Beijing type (8%), as the most common. Eighteen spoligotypes did not match any existing database pattern.

India accounts for 30% of tuberculosis (TB) cases worldwide. Each year, this disease develops in approximately 2 million people in India. Approximately 0.5 million people die, a figure likely to increase with emergence of multidrug-resistant tuberculosis (MDR-TB) and the HIV epidemic (*1*). Delhi alone has an annual risk for infection of 2.4% (state TB officer, Delhi, pers. comm.). Traditional methods for tracing transmission of TB are imprecise and ineffective in controlling the disease. The impact of control programs is often difficult to assess in high-incidence areas, where infection and disease patterns are highly heterogeneous. This difficulty can be overcome by an alternative approach in which molecular strain-typing techniques are used. Spoligotyping detects the presence and absence of nonrepetitive unique spacer sequences (36–41 bp in length) in the direct repeat region of *Mycobacterium tuberculosis*. Its utility as an initial screening method is well documented (*2**–**4*).

The aim of our study was to identify predominant spoligotypes with an international designation responsible for transmission and prevalence of TB in Delhi. The spoligotypes obtained were compared with an international spoligodatabase, spolDB3.0 (5,6).

## The Study

The study included patients with culture-confirmed TB whose cases were reported to a district TB center, a primary health center, and an outpatient department, All India Institute of Medical Sciences (AIIMS), Delhi, the coordinating center. AIIMS is the top tertiary-care hospital with referrals from the entire city of Delhi, as well as from other states, with a daily outpatient attendance of 4,000 to 5,000. The district TB and primary health centers serve a well-defined population, including all TB patients in their areas.

Patients with a diagnosis of new, smear-positive pulmonary TB or those with a high suspicion for TB on clinical or radiologic grounds were included in the study. In all, 1,500 patients who met inclusion and exclusion criteria were recruited from the Delhi area over 2 years. Demographic data were collected on the patient's sex, age, present address, employment, economic status, literacy, living conditions, household contacts, chest radiologic findings, and HIV infection. Three sputum specimens were collected in the early morning on consecutive days and transported to the AIIMS TB laboratory. Sputum specimens were processed by Petroff's method and plated on duplicate Lowenstein-Jensen slants. A smear was examined after Ziehl-Neelsen staining. Species confirmation was followed by tests for drug susceptibility (proportion method) for rifampicin (RIF, 40.0 mg/mL), ethambutol (ETH, 2.0 mg/mL), streptomycin (STR, 4.0 mg/mL), and isoniazid (INH, 0.2 mg/mL) (*7*). The isolates were processed on the respective days of collection with the date and other details recorded. During the initial 3 months, January 2001–March 2001, 105 (55%) of 190 patients enrolled were culture-positive. The 105 individual *M. tuberculosis* isolates from different patients (34 from the district center, 12 from the primary health center, and the rest from AIIMS), both smear-negative and smear-positive samples, were spoligotyped. Work on the isolates collected later in the study is ongoing.

Spoligotyping to detect 43 known spacers in the direct repeat locus was performed with a commercially available kit, according to the instructions supplied by the manufacturer (Isogen Bioscience B.V., Maarsen, the Netherlands). To avoid any possibility of artifactual hybridization spots on the commercial membranes, appropriate controls included DNA from *M. bovis* and *M. tuberculosis* H37Rv and autoclaved purified water for adequate number of negative controls in each experiment. Reproducibility of spoligotyping was confirmed by repeating the test with the DNA extracted again from a few isolates (data not shown). None of the negative controls demonstrated carryover DNA.

Results were doublechecked visually by an experienced operator to eliminate any systematic artifact caused by using commercial membranes. The results obtained were entered in a binary format as Excel spreadsheets (Microsoft, Redmond, WA) and compared to the spolDB3.0 of the Pasteur Institute of Guadeloupe. At the time of the matching analysis, spolDB3.0 contained 13,008 patterns distributed into 813 shared types (patterns reported at least twice that grouped 11,708 clinical isolates) and 1,300 orphan patterns from >90 countries (*6*). The results were also computed into Recognizer files of the Taxotron package (P.A.D. Grimont, Taxolab, Institut Pasteur, Paris) to calculate the 1-Jaccard Index (*8*) and to allow the construction of dendrograms by using the unweighted pair-group method with arithmetic averages (UPGMA [9]). Odds ratios for clustering with 95% confidence intervals were calculated to compare characteristics of clustered and nonclustered patients. Differences were considered significant if values were <0.05 (Table 1).

**Table 1:** Clinical and epidemiologic characteristics of patients harboring clustered versus nonclustered strains^a^

Parameters	No. (%) of patients in			
	Clustered group	Nonclustered group	OR for clustering (95% CI)	p value
Age, y				
15–45	63 (81)	15 (19)	4.52 (1.6 to 12.96)	0.001
>46	13 (48)	14 (52)		
Sex				
Male	53 (72)	21 (28)	0.88 (0.30 to 2.49)	NS
Female	23 (74)	8 (26)		
HIV status				
Seropositive	2 (100)	0 (0)	UD	
Seronegative	74 (72)	29 (28)		
Previous history of TB				
No previous therapy	52 (80)	13 (20)	2.53 (0.89 to 7.22)	0.05
Previously treated	19 (61)	12 (39)		
Drug resistance				
Drug resistance^b^	22 (71)	6 (29)	1.56 (0.51 to 4.97)	NS
Susceptible to all drugs	54 (70)	23 (30)		
Radiologic findings				
Extensive cavitary	12 (76)	5 (24)	1.6 (0.33 to 11.22)	NS
Limited cavitary	6 (60)	4 (40)		
Sputum smear positive				
1–10 AFB/10–100 fields	30 (73)	11 (27)	0.96 (0.34 to 2.70)	NS
>1 AFB per field	37 (74)	13 (26)		

^a^OR, odds ratio; CI, confidence interval; TB, tuberculosis; NS, not statistically significant; UD, undefined; AFB, acid-fast bacilli. ^b^Resistance to one or more drugs.

Table 1 shows the detailed demographic data of the study population. None of the patients' families had symptoms suggestive of TB during the study period. In 2% of families, death attributable to pulmonary TB was reported.

A total of 45 distinct spoligopatterns were obtained from the study population of 105 isolates (Figure). Twenty-nine (28%) clinical isolates were represented by a unique pattern, whereas 76 (72%) isolates were clustered in 16 clusters, i.e., 2 predominant clusters of 23 (22%) and 13 (12%) isolates (ST26 and ST54), followed by 1 cluster of 9 isolates (ST1), 2 clusters of 4 isolates each (ST11 and ST119), 1 cluster of 3 isolates (ST1088), and 10 clusters of 2 isolates (ST100, ST276, ST1089-1092 and ST1094-1097). The isolates in all the clusters had different drug-resistance profiles and had been collected, processed, and amplified into separate batches on different days; hence, carryover contamination was ruled out.

**Figure:**
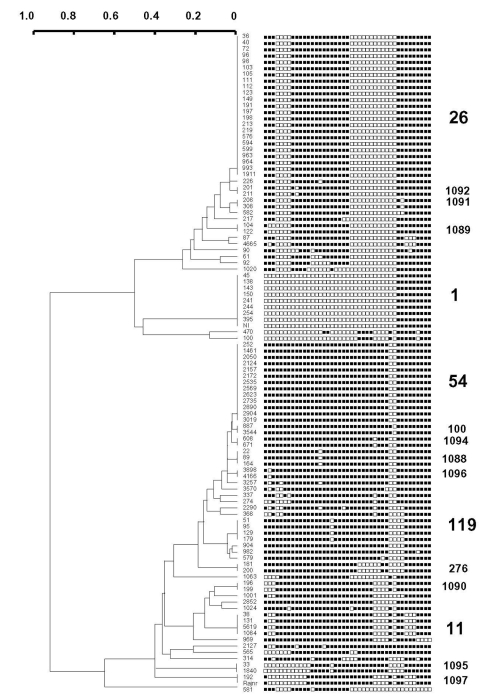
Dendrogram built on 105 *Mycobacterium tuberculosis* clinical isolates from Delhi based on spoligotyping results using the Taxotron software package (PAD Grimont, Taxolab, Institut Pasteur, Paris). This phylogenetic tree, based on the 1-Jaccard Index (*8*) and drawn using the unweighted pair group method with arithmetic averages (UPGMA), shows the presence of five major shared types (ST) of spoligotypes in Delhi; ST26 (Central Asian Family 1 or CAS1), ST1 (Beijing Family), ST54 (also newly designated as T1 ancestor), ST119 (X1 family) and ST11 (East African Indian 3 or EAI3 family). The strain designation appears left of the binary spoligotyping profile, and shared-type designations appear on the right of the spoligotype. Orphan patterns (unique isolates) have not been provided with a spoligotype designation on this tree.

Spoligotypes were compared with those in spolDB3.0. The three most prevalent spoligotypes from this study were type 26, 54, and 1. ST26 (22%) was initially described in 1997 in a study performed in the United Kingdom (*4*). It was later shown to belong to the major genetic group I of *M. tuberculosis* complex organisms (*10**,**11*), i.e., an ancestral group of human TB, as evidenced by the presence of the TbD1 region in these strains (*12*). Until now, this family of strains has been reported in 11 countries of the Middle East (Iran, Pakistan, and India), Oceania (Australia), the United States, and Europe (United Kingdom, the Netherlands, France, Sweden, Italy, and Austria [5]; ). In Europe and Australia, these strains were regularly found to be linked with immigrants from the Middle East and Central Asia, hence the name of Central Asian 1 or CAS1 family, which was recently given to all isolates characterized by the absence of spacers 4–7 and 23–34 (*6*). Indeed, a total of 20 shared-type variants linked to this family of strains have been found to date (*5*). In a recent article, this predominant clade of bacilli and some of their variants were called the Delhi type (*13*). For spoligotype patterns, see Table 2.

**Table 2:** Predominant spoligotype prevalent in Delhi and shared type designations for 10 unique isolates, clade designation, and initial description (reference)

Spoligotype designation	Spoligotype pattern	Clade^a^	Reference
Predominant spoligotype			
Type 26	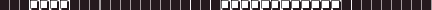	Casi (Delhi)	13
Type 54	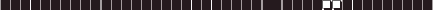	Manu	5
Type 1	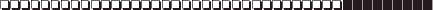	Beijing	10
Shared spoligotype			
Type 50	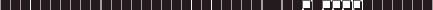	H3	4
Type 52	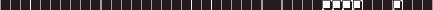	T2	4
Type 53	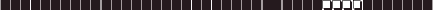	T1	4
Type 138	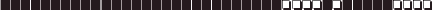	Eai	4
Type 141	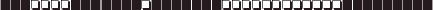	Cas1	5
Type 357	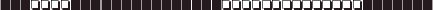	Cas1	5
Type 381	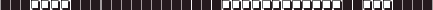	Cas1	4
Type 427	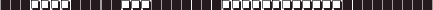	Cas1	5
Type 458	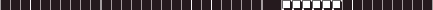	Eai	5,14
Type 1093	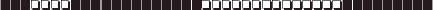	Cas	4

^a^Clades were defined according to the definitions in spolDB3.0 (*6*); = no hybridization, = positive hybridization. CAS1 (Central Asian 1) family (also termed as the "Delhi type") is characterized by 4–7 and 23–34; Manu (derived from the name of a Hindu mythological figure supposed to be the world's first king and father of the human race) is characterized by 33–34 (*5*), and presumed to be the probable ancestor of both the CAS and EAI (East African Indian). Beijing family is characterized by 1–34. Both EAI and CAS1 belong to the Major Genetic Group 1; H3 (Haarlem 3) belongs to the Major Genetic Group 2; and T1 and T2 are poorly defined families that belong to the Major Genetic Group 2 or 3 (*11*). T1 and T2 need other markers for a better characterization (T1, 33–36 and T2, 33–36 and 40 [6]).

Type 54 (12%) is also likely to belong to group I organisms, as suggested by the existence of a closely linked profile, found within the Houston study (clinical isolate S179; [10]). This shared-type is less widespread, and its distribution is different. It has been reported to be present in Africa (Guinea-Bissau and Senegal) and in Europe (France, United Kingdom, and the Netherlands). This type may be an ancestor of both the CAS and the Beijing family. It is characterized by the absence of spacers 33 and 34, two spacers likely to be of high phylogenetic importance in group I organisms since they permit distinguishing between 1) East African Indian (EAI) superfamily (presence of spacer 33, absence of 34), 2) CAS family (absence of 33 and 34), and 3) *M. bovis* (presence of 33 and 34). Combined prevalence of these two spoligotypes (34%) indicates that these two families are highly prevalent in our high-incidence area and may play an important role in disease transmission in Delhi (Table 2).

The third most prevalent type is the Beijing type shown in nine isolates (8%). This type was originally described by van Soolingen et al. in China and is highly prevalent throughout Asia and Eurasia (*15*), with a reported prevalence of approximately 3% in India (*13*). Among these isolates, seven were resistant to ETH, STR, and INH, and one isolate (strain 45) was resistant to all four drugs tested. An epidemiologic link could be established for eight patients who resided in one area (Faridabad) and were referred to the district TB center. Two shared types (ST11 and ST119) belong to the EAI and X families, respectively (*14*). The presence of the X family in India could be linked to the past British history in this region.

Spoligotypes that did not match any existing pattern in the database were defined as orphans. Of 45 patterns observed in this study, 18 (observed for isolates 4665, 90, 92, 1020, 470, 100, 3257, 3570, 337, 274, 2290, 368, 1063, 1024, 2127, 565, 314, 581) were true orphans (no counterpart in the database). This percentage referred to patterns (40%) is high and reflects both the current absence of knowledge on the genetic diversity of Indian *M. tuberculosis* strains and the microevolutionary genetic driving forces active in TB-epidemic dynamics in India. Of 105 isolates, 18 (17%) had orphan spoligopatterns. New shared types were also created for newly identified types (ST1088–1092 and 1094–1097), which may either reflect homoplasia (creation of common genetic structures without common ancestor, also called convergence [16]; ) or true synapomorphy (common ancestors). Except for one clinical isolate belonging to ST1092, isolated in 2002 in New York (S863, J. Driscoll, unpub. data), all the shared types mentioned above have not been reported elsewhere in the world. A total of 10 isolates unique to this study (Table 2) did match with strains already reported elsewhere (ST50, 52, 53, 138, 141, 357, 381, 427, 458, and 1093). The origin of their counterparts from different parts of the world is described in spolDB3.0. However, many of these patterns (particularly ST 138, 381, 1093) were originally reported in the United Kingdom by Goyal et al. in 1997 (*4*), who described many strains harboring Indian genetic characteristics.

Little information is available from India or the neighboring countries on the molecular epidemiology of TB. Our study has demonstrated that the epidemiology of TB in India is much different than TB epidemiology elsewhere (*5*). Our results showed significant clustering in the 15- to 45-year age group (p = 0.001) and significant lack of clustering in the older age groups. Patients with no history of previous treatment also showed significant clustering (p = 0.05). No significant differences were observed for other parameters such as age <60 years, gender, HIV status, drug resistance, radiologic findings, and sputum smear positivity.

## Conclusions

Spoligotyping is based on the variability in the direct repeat locus of *M. tuberculosis*, which most likely occurs by one of three mechanisms—homologous recombination between neighboring or distant direct variable repeats, IS-mediated transposition, and DNA replication slippage (*17*). Spoligotyping is useful for tracking TB epidemics, detecting new outbreaks, and better defining high-risk populations to focus prevention strategies (*6**,**18*). Spoligotyping may also constitute a potential tool for global TB epidemiology, population genetics, and phylogeny, although it should be used with another independent genotyping method in many settings to prove clonality (*5**,**6**,**10*). Another poorly investigated theoretical limitation issue is the level of convergence that may jeopardize phylogenetic reconstruction when spoligotyping is used (*16*).

To have a better knowledge of moving and expanding clones of *M. tuberculosis* within the Indian subcontinent, we attempted to identify the predominant spoligotypes prevalent in Delhi and determine their specific signature. Comparison with the spolDB3.0 database enabled us to compare spoligotypes generated in a New Delhi laboratory with different laboratories around the world. The present study included few isolates from patients enrolled at one center, in the beginning of a multicentric study, with no bias for patient selection; hence, it was truly representative of the community. As a a tertiary-care referral center, AIIMS gets patients from throughout Delhi. In addition, isolates from the district TB and the primary health centers (which cover all the patients in their respective areas) were also included. It seemed imperative to study the population structure of Indian *M. tuberculosis* strains by using additional genetic markers to better comprehend the origin and evolutionary genetics of TB in this geographic region. The study strains have been further subjected to IS*6110-*RFLP and double repetitive element PCR. Also the spoligo clusters are being subjected to mycobacterial interspersed repetitive units—variable number of DNA tandem repeats. Investigation of strains from Delhi and those collected from the other centers from all over the country is ongoing.

Clustering was much higher within the age group of 15 to 45 years but decreased with increasing age and was lowest in patients >60 years (odds ratio for clustering 4.52, p = 0.001). This finding may suggest active transmission of TB among younger persons as opposed to possible reactivation of disease in the older age group. A high rate of clustering in a population with no history of TB treatment further corroborated the notion of active transmission of certain prevailing genotypes, despite the existence of genetically diverse strains in our high-incidence community. These prevailing genotypes may play an important role in the propagation of the TB epidemic in Delhi and surrounding regions in coming years. Extensive cavitary disease and degree of infectiousness (smear positivity) indicative of large bacterial populations did not affect clustering.

Studies focusing on the polymorphism of *M. tuberculosis* isolates from developing countries, where TB is highly prevalent, would provide new insights into epidemiology, transmission dynamics, phylogenetic analysis, and virulence. Similar studies with detailed epidemiologic data that would reflect on the TB control programs are needed to understand the current epidemic in India.

## References

[1] Centers for Disease Control and Prevention. Progress toward tuberculosis control—India. MMWR Morb Mortal Wkly Rep. 2001;51:229–32. 11925019

[2] Kremer K, van Soolingen D, Frothingham R, Haas WH, Hermans PWM, Martin C, Comparison of methods based on different molecular epidemiological markers for typing of *Mycobacterium tuberculosis* complex strains: interlaboratory study of discriminatory power and reproducibility. J Clin Microbiol. 1999;37:2607–18. 10405410 10.1128/jcm.37.8.2607-2618.1999 PMC85295

[3] Kamerbeek J, Schouls L, Kolk A, van Agterveld M, van Soolingen D, Kuijper S, Simultaneous detection and strain differentiation of *Mycobacteium tuberculosis* for diagnosis and epidemiology. J Clin Microbiol. 1997;35:907–14. 9157152 10.1128/jcm.35.4.907-914.1997 PMC229700

[4] Goyal M, Saunders NA, van Embden JDA, Young DB, Shaw RJ. Differentiation of *Mycobacterium tuberculosis* isolates by spoligotyping and IS*6110* restriction fragment length polymorphism. J Clin Microbiol. 1997;35:647–51. 9041405 10.1128/jcm.35.3.647-651.1997 PMC229643

[5] Filliol I, Driscoll JR, van Soolingen D, Kreiswirth BN, Kremer K, Valétudie G, A snapshot of moving and expanding clones of *Mycobacterium tuberculosis* and their global distribution assessed by spoligotyping in an international study. J Clin Microbiol. 2003;41:1963–70. 10.1128/JCM.41.5.1963-1970.2003 12734235 PMC154710

[6] Sola C, Filliol I, Guttierez C, Mokrousov I, Vincent V, Rastogi N. Spoligotype database of *Mycobacterium tuberculosis*: biogeographical distribution of shared types and epidemiological and phylogenetic perspectives. Emerg Infect Dis. 2001;7:390–6. 10.3201/eid0703.010304 11384514 PMC2631784

[7] Laszlo A, Rahman M, Raviglione M, Bustreo F. WHO/IUALTD Network of Supranational Reference Laboratories. Quality assurance programme for drug susceptibility testing of *Mycobacterium tuberculosis* in the WHO/IUALTD Supranational Laboratory Network: first round of proficiency testing. Int J Tuberc Lung Dis. 1997;1:231–8. 9432369

[8] Jaccard P. Nouvelles recherches sur la distribution florale. Bull Soc Vaud Sci Nat. 1908;44:223–70.

[9] Sneath PH, Sokal AR. Numerical taxanomy: the principles and practices of classification. San Francisco: W.H. Freeman and Co.; 1973.

[10] Soini H, Pan X, Amin A, Graviss EA, Siddiqui A, Musser JM. Characterization of *Mycobacterium tuberculosis* isolates from patients in Houston, Texas, by spoligotyping. J Clin Microbiol. 2000;38:669–76. 10655365 10.1128/jcm.38.2.669-676.2000 PMC86172

[11] Sreevatsan S, Pan X, Stockbauer K, Connell N, Kreiswirth B, Whittam T, Restricted structural gene polymorphism in the *Mycobacterium tuberculosis* complex indicates evolutionarily recent global dissemination. Proc Natl Acad Sci U S A. 1997;97:9869–74. 10.1073/pnas.94.18.9869 9275218 PMC23284

[12] Brosch R, Gordon SV, Marmiesse M, Brodin P, Buchrieser C, Eiglmeier K, A new evolutionary scenario for the *Mycobacterium tuberculosis* complex. Proc Natl Acad Sci U S A. 2002;99:3684–9. 10.1073/pnas.052548299 11891304 PMC122584

[13] Vijaya-Bhanu N, van Soolingen D, van Embden JDA, Dar L, Pandey RM, Seth P. Predominance of a novel *Mycobacterium tuberculosis* genotype in the Delhi region of India. Tuberculosis (Edinb). 2002;82:105–12. 10.1054/tube.2002.0332 12356462

[14] Sebban M, Mokrousov I, Rastogi N, Sola C. A data-mining approach to spacer oligonucleotide typing of *Mycobacterium tuberculosis.* Bioinformatics. 2002;18:235–43. 10.1093/bioinformatics/18.2.235 11847071

[15] van Soolingen D, Qian L, de Haas PEW, Douglas JT, Traore H, Portaels F, Predominance of a single genotype of *Mycobacterium tuberculosis* in countries of east Asia. J Clin Microbiol. 1995;33:3234–8. 8586708 10.1128/jcm.33.12.3234-3238.1995 PMC228679

[16] Warren RM, Streicher EM, Sampson SL, van der Spuy GD, Richardson M, Nguyen D, Microevolution of the direct repeat region of *Mycobacterium tuberculosis*: implications for interpretation of spoligotyping data. J Clin Microbiol. 2002;40:4457–65. 10.1128/JCM.40.12.4457-4465.2002 12454136 PMC154636

[17] van Embden JDA, van Gorkom T, Kremer K, Jansen R, van der Zeijst BAM, Schouls LM. Genetic variation and evolutionary origin of the direct repeat locus of *Mycobacterium tuberculosis* complex bacteria. J Bacteriol. 2000;182:2393–401. 10.1128/JB.182.9.2393-2401.2000 10762237 PMC111299

[18] Quitugua TN, Seaworth BJ, Weis SE, Taylor JP, Gillette JS, Rosas II, Transmission of drug-resistant tuberculosis in Texas and Mexico. J Clin Microbiol. 2002;40:2716–24. 10.1128/JCM.40.8.2716-2724.2002 12149319 PMC120686

